# Genetic Testing for Minors: Comparison between Italian and British Guidelines

**DOI:** 10.1155/2012/786930

**Published:** 2012-03-06

**Authors:** Pamela Tozzo, Luciana Caenazzo, Daniele Rodriguez

**Affiliations:** Department of Environmental Medicine and Public Health, Legal Medicine Unit, University of Padua, Via Falloppio 50, 35121 Padua, Italy

## Abstract

Genetic testing in children raises many important ethical, legal, and social issues. One of the main concerns is the ethically inappropriate genetic testing of minors. Various European countries established professional guidelines which reflect the different countries perspectives regarding the main ethical issues involved. In this paper, we analyze the Italian and the British guidelines by highlighting differences and similarities. We discuss presymptomatic, predictive, and carrier testing because we consider them to be the more ethically problematic types of genetic testing in minors. In our opinion, national guidelines should take into account the different needs in clinical practice. At the same time, in the case of genetic testing the national and supranational protection of minors could be strengthened by approving guidelines based on a common framework of principles and values. We suggest that the Oviedo Convention could represent an example of such a common framework or, at least, it could lead to articulate it.

## 1. Introduction

Genetic testing of minors raises many important ethical issues [1–4]. It can often generate information about future impairments in children's health without helping physicians to establish diagnostic and/or therapeutic processes. First of all, we should consider the impact of genetic tests on the child: they may lead to stigmatization and they could change the day-to-day family's life. Moreover, the child may also feel depressed or worried about the possible consequences of his/her genetic disorders, especially when it is not clear how they will affect their future [5, 6]. Another important issue concerns the child's right not to know, by considering that minors have the right to participate in the medical decision-making process.

In Article 12 of the United Nations “Convention on the Rights of the Child” we read that a “child who is capable of forming his or her own views [has] the right to express those views freely in all matters affecting the child, the views of the child being given due weight in accordance with the age and maturity of the child” [7]. It is important to bear in mind that, in the case of genetic testing, as well as in the case of similar issues in medicine, the child's privacy and confidentiality need to be respected [8–10].

In many countries, national guidelines have been developed. They address various aspects of childhood testing [1, 2, 11]. In our opinion, it could be interesting to question whether there are any national cultural factors or particular policies which could contribute to approach the topic differently.

Genetic information challenges the individualistic nature of many moral assumptions made in discussing issues in medical ethics, because of the familial nature of much genetic data. On the one hand, in the UK, the Human Genetics Commission was established partly in response to the public intuition that genetic information is especially private: its more recent work [12] reflects both scientific developments in genetics and the policy pressures to make ever more use of genetic information. In particular, issues include the increasing preimplantation genetic diagnoses, direct-to-consumer genetic testing, and government plans to expand the national DNA database for forensic purposes.

On the other hand, in Italy, where the historical and cultural heritage of Catholicism is an integral element of national identity and where the multicultural approach to medical issues is increasing, great importance is given to the social, religious, and familial implication of genetic counseling. Furthermore, we notice the focus on reducing the burden of disease and disability, on self-determination, and on living in a just and inclusive society [13].

In this paper, we study these two different approaches by considering two guidelines as examples. They are the Guidelines for the “Genetic Testing of Children” of the British Society for Human Genetics (BSHG) [14] and the “Guidelines for Medical Genetics Activities” of the Italian Permanent Conference for Relations between the State and the Regions and Autonomous Provinces of Trent and Bolzano [15]. We focus our attention on presymptomatic, predictive, and carrier testing because they raise major ethical concerns.

Carrier tests are used to determine whether an individual carries a mutated gene or a balanced chromosomal rearrangement. Two are the main ethical issues concerning carrier tests: first, the information about carrier status does not provide immediate medical benefit to the minor who is tested; second, this type of testing may violate the minor's rights of privacy, confidentiality, and autonomous decision-making in adulthood [11].

Presymptomatic and predictive genetic testing refers to the possibility of finding a genetic alteration before the appearance of symptoms related to that alteration. Testing young people for adult-onset disorders is the most controversial predictive testing because we do not know yet any treatment that could prevent the onset and progression of the disease [16]. In this case, if the test is predictive and there are no direct or immediate medical benefits available, we are left only with the possibility of deciding whether to perform a genetic test on a minor and, in the affirmative, when and how we should perform it.

For the purposes of the present paper, we use “minor” and “child” as synonymous, according to Article 1 of the United Nations “Convention on the Rights of the Child” [7] which states that “a child means every human being below the age of eighteen years unless, under the law applicable to the child, majority is attained earlier” and considering that, both in Italy and in the UK, majority is reached at 18 years old.

## 2. Materials and Methods

We analyze the Guidelines for the “Genetic Testing of Children” of the BSHG and the document entitled “Guidelines for Medical Genetics Activities” of the Italian Conference State-Regions because they are two ethically relevant documents. In particular, we highlight both their common and different ethical choices.

The 2010 BSHG report was prepared by professionals working in human genetics in the UK. It revisited the issues explored in 1994 by the Clinical Genetic Society in light of subsequent developments and of the other guidelines published since 1994.

The Italian guidelines were approved in 2004 by the Permanent Conference for Relations between the State and the Regions and Autonomous Provinces of Trent and Bolzano. This Conference is an important body where a continued dialogue is maintained between the State and Regions on various health-care issues in relation to the whole system of local self-government; it also facilitates cooperation between central government, local authorities, and regions. The Italian guidelines were set up to provide common and harmonized national directives in medical genetics. They aimed at assuring to Italian citizens the benefits of proper levels of assistance and quality in performing genetic tests.

## 3. Results and Discussion

According to both guidelines, the ethically more relevant aspects in performing genetic testing on minors are the timing of performing predictive or presymptomatic tests, the possibility of performing carrier testing, and the involvement of the minor in the decision-making process by helping him or her to understand the meaning and the purpose of the test results.

One of the main controversial issues is the capacity to perform predictive or pre-symptomatic testing on asymptomatic minors. The BSHG guidelines recommend that “in such circumstances testing should normally be delayed until the young person can decide for him/herself when, or whether, to be tested” [14]. They also state that “this does not mean that childhood testing for such conditions should never be done” [14]. They justify their recommendation by explaining that testing in childhood may afflict future autonomous decisions. It is also recommended that, when a parent requests to have a child tested and this test has no direct or immediate medical benefits, “an assessment should be made of the balance of harms and benefits” [14] by considering the child's best interests as the basis of the decision-making process.

Paragraph 4.1 of the Italian guidelines is entitled “Tests on Minors.” There we read that presymptomatic tests should be deferred until the minors reach legal maturity (i.e., 18 years old in Italy). These genetic tests, however, can be performed on a minor with the consent of both parents (or of the legal guardian) when there are current and concrete therapeutic or preventive possibilities. In this paragraph, great attention is paid to the psychological consequences of this type of testing, such as the effects on the minor's self-esteem, the possible changes in the parent-child relationship, the stigmatization of healthy brothers or sisters, the stigmatization of the minor in the educational setting, and the possible consequences on his/her future career, as well as emotional implications.

With respect to this topic, we conclude that, both in Italy and in the UK, these guidelines recommend that the availability of therapeutic or preventive measures be a prerequisite for testing asymptomatic minors, regardless of age. Tests with no current benefits for children are to be deferred until the minor is 18 years old.

Regarding any genetic condition which is likely to occur during childhood, the BSHG guidelines suggest a cautious approach. There may be good reasons to defer testing, while in the Italian guidelines there are no specific recommendations on this aspect.

Regarding genetic testing to establish if a minor is carrier of a specific gene, the Italian guidelines discourage only prenatal tests by considering that, in the vast majority of cases, being a carrier of an autosomal-recessive condition or a female carrier of an X-linked recessive mutation has no implications for the child's health (other than related to future reproductive choices). But testing is accepted when both parents are carriers of the gene causing a specific disease and when there is the concrete possibility that their child carries that disease.

In the BSHG guidelines, carrier testing is examined in Part C by discussing a clinical case (no. five). The scenario is the following: a couple had their child tested with a newborn screening program for cystic fibrosis (CF)—an autosomal recessive condition in which affected children carry two altered copies of the CF gene. The father and the baby are found to be carriers, while the mother is not. The couple has two other children, aged 6 and 8 years old. They request their children to be tested and learn that they are also carriers. The ensuing discussion focuses on the following aspects: “testing for carriers of sex-linked and chromosomal disorders is often a weightier matter than for autosomal recessive disease…it is appropriate for professionals to consider the timing of testing for carrier status of sex-linked and chromosomal disorders rather more carefully than for most autosomal recessive conditions” [14]. Moreover, “it is important that the young person is adequately informed about their reproductive risks and we believe this is more likely to be the case if they themselves are closely involved in any decision about genetic testing” [14]. Thus, carrier testing is allowed, especially for X-linked or chromosomal disorders, whether or not the test is performed on minors. Health-care professionals and genetic counselors are expected to support families in discussing the situation with the child as he/she grows up and reaches maturity.

In the BSHG guidelines, great importance is given to discussing with “all relevant parties” [14] the timing of predictive testing. But the identity of these relevant parties is not specified: they might be the child's parents, but, in the case of genetic information, other family members could be involved. Hence, health-care professionals are left with uncertainty about who should be involved in the decision-making process. In particular, point 6 of Part A states that health-care professionals should facilitate “discussions within the family” [14], without specifying the meaning of the word “family” in this context.

Paragraph 2 of the Italian guidelines affirms that “The process of genetic counseling aims at helping the person and the family” [15] to understand all the medical information regarding the test and its possible consequences, without specifying the role of minors in this information process. Yet, the paragraph notes that the process of genetic counseling may have important ethical and psychological consequences for minors, particularly related to reproductive choices and to the possibility for the minor of knowing or not knowing his/her genetic features and their future effects. In this case, it seems that no attention is given to people who cannot make autonomous decisions and who might not be well aware of the consequences of a specific genetic mutation, such as young children. This approach is representative of the lack of attention in the Italian health-care setting to inform minors and to promote their role in the decision-making process. Hence, we stress the need for more binding provisions and for a better cooperation among health-care professionals.

When we reflect on genetic information, the BSHG guidelines emphasize the importance of parents talking to their children about their family history from a young age. We believe that this could lead to controversial situations in daily family life. Sometimes, it might be difficult to explain to one's child the “family history.” At an earlier stage of development, this history might influence negatively the child's development. It could be advisable to wait until the minor is older.

According to paragraph 3.3 of the Italian guidelines, in case of minors the genetic information should be given only to the parents. To them, it should also be specified “the reason why the test can be helpful, the pros and cons of the test, the possible limits of the results, and the implications for the patients and other family members”. Additionally, it is important to ensure the patient's capability of making autonomous decisions on the basis of his/her values. Minors, however, might not be able to make autonomous decisions because their whole set of values is not yet fully established. Their role in the decision-making process might not be proactive. Paragraph 7.2 affirms that genetic counseling is of great importance for patients who cannot read or cannot understand written documents. Is it possible to extend this provision to children who do not yet read or to minors with developmental or speech impairment?

Finally, in either set of guidelines it is not clear how the decision-making process should be performed when those with responsibility of caring for the child cannot come to an agreement. This might occur frequently in daily practice, especially when we remember that discovering about one's child genetic mutations might increase the parents' anxiety, depression, and suffering; it could also lead to conflicts between parents concerning how to proceed.

## 4. Conclusion

Decisions about the timing of genetic testing raise different difficulties for parents, children, and health-care professionals. Regarding predictive tests, in order to protect the privacy and confidentiality of genetic information and the minor's right not to know, the British and Italian guidelines suggest to postpone testing asymptomatic children, when there is no urgent medical need. The BSHG guidelines support testing when the minor can participate in the decision-making process, while the Italian guidelines propose that this type of test be deferred until the minor reaches the age of 18 years old.

Carrier testing is approached differently in both guidelines. The Italian document suggests that this kind of analysis should not to be performed, except in the case of prenatal diagnosis when both parents are carriers and when there is a relevant risk for the child to be affected. The British approach is quite different: carrier testing could be performed, especially for X-linked and chromosomal disorders, and health-care professionals should facilitate the family's decision-making process.

Both guidelines lack details about the role of minors in the decision-making process. Health-care professionals are left on their own to form their own judgments in light of their values, by deciding who, among the family members, should be involved, whether or not directly inform the minor, how and when to inform the child. Moreover, British guidelines give importance to the communication process between parents and minors, while Italian provisions emphasize the role played by health-care professionals and parents. It is interesting to notice that neither in the BSHG guidelines nor in the Italian ones there are recommendations about how to address possible conflicts between those entitled to care for the child.

One could argue that differences in national professional guidelines can enrich the debate and respond to specific and peculiar instances in the day-by-day clinical practice. Nevertheless, we think that these differences and similarities in attitudes between the Italian and British professional guidelines should be further examined to ensure the protection of vulnerable people in the European context. In our opinion, in developing national guidelines on genetic testing, we should take into account differences in clinical practice, but a common effort is needed to assure the protection of minors. Hence, the approval and the application of national guidelines should be promoted. These guidelines should also express a common framework of principles and values.

As a concluding example, regarding information and consent in medical treatments involving minors, we believe it is interesting to underline the importance of the “Convention for the Protection of Human Rights and Dignity of the Human Being with regard to the Application of Biology and Medicine” [17]. This Convention was adopted by the Committee of Ministers of the Council of Europe in November 1996 and it was opened for signature in Oviedo (Spain) on 4 April 1997. It aims at providing a common framework for the protection of human rights and human dignity in biology and medicine. In article 6, the Convention establishes that “Where, according to law, a minor does not have the capacity to consent to an intervention, the intervention may only be carried out with the authorization of his or her representative or an authority or a person or body provided for by law.” Moreover, “The opinion of the minor shall be taken into consideration as an increasingly determining factor in proportion to his or her age and degree of maturity” [17].

We think that the Convention focus on rights and human dignity, as well as its specific proposals, could be useful to promote dialogue and interaction between different cultural approaches, such as the two analyzed in this article, even being aware that the UK is not a signatory of the Oviedo Convention. In conclusion, we acknowledge the ethical complexity of deciding on the timing of genetic tests in the case of minors. We emphasize our desire for articulating procedural guidelines—national and supranational—and for their implementation to help us in practicing genetic medicine within our society.
